# Osteosarcoma of the Maxilla: A Rare Case with Unusual Clinical Presentation

**DOI:** 10.5681/joddd.2013.029

**Published:** 2013-08-30

**Authors:** Pouyan Amini Shakib, Ramin Foroughi, Maryam Seyedmajidi

**Affiliations:** ^1^Assistant Professor, Department of Oral and Maxillofacial Pathology, Faculty of Dentistry, Babol University of Medical Sciences, Babol, Iran; ^2^Assistant Professor, Department of Oral and Maxillofacial Surgery, Faculty of Dentistry, Babol University of Medical Sciences, Babol, Iran; ^3^Assistant Professor, Dental Materials and Research Center, Babol University of Medical Sciences, Babol, Iran

**Keywords:** Osteosarcoma, maxilla, bone tumor

## Abstract

Osteosarcoma (OS) is a malignant mesenchymal tumor, which rarely occurs in the maxilla. Although variable histologic and radiographic features of OS have been reported previously, in the majority of the cases painful swelling of the jaw is mentioned as the first clinical presentation. Furthermore, early diagnosis and wide surgical resection of the tumor are the most important determinant factors of prognosis. Therefore, the unusual clinical presentations of OS should be considered meticulously to expedite the diagnosis process. We describe a case of OS of the maxilla with extremely unusual presenta-tion in a 42-year-old female, that was initially designated as “epulis fissuratum”. Here, we highlight the importance of com-bining the clinical, radiographic and histopathologic examination to obtain a definitive diagnosis and also the significance ofearly effective surgical intervention in evaluation of pathologic lesions.

## Introduction

Osteosarcoma (OS) is the most common primary malignant bone tumor; however, only 5% of all cases occur in the jaws. The maxilla is less commonly affected than the mandible and in the maxilla the majority of OSs arises in the alveolar ridge and the maxillary antrum. Symptoms usually include painful swelling in the area and loosening of teeth, although paresthesia, nasal obstruction and ophthalmic complications such as proptosis may be noted.^1^ In addition, unusual clinical presentation such as periapical lesion-like appearance has been reported.^2^

The management of all variants of osteosarcoma, including low-grade, intramedullary, and juxtacortical osteosarcomas, are identical with complete surgical excision of the tumor together with wide safe surgical margins. Although other treatment modalities such as chemotherapy and radiation therapy have been proposed, the majority of the tumors are clinically identified in advanced stages and require combination treatment.^1,3^ Hence, early diagnosis, especially in OSs with unusual clinical presentations, has a great impact on treatment planning and prognosis of the patients.

The authors would like to report a case of OS of the maxilla with extremely unusual presentation in a 42-year-old woman and also describe a comprehensive evaluation of the patient, which led to definitive diagnosis and early effective treatment.

## Case report

A 42-year-old female patient was referred to the Faculty of Dentistry of Babol University of Medical Sciences by a dentist due to dull pain on the left side of her face.

### Physical Examination

On the clinical examination the patient, who had been wearing an upper denture for about 5 years, reported a dull pain since two months earlier on the left side of her face, which was attributed to the irritation of the prosthetic appliance fabricated by the general practitioner, who had examined the patient previously and made a diagnosis of “epulis fissuratum”.

Extra-oral examination was unremarkable, no palpable lymph node at any level of the head and neck region was detected and no contributory past medical history was noted.

**Figure 1:**
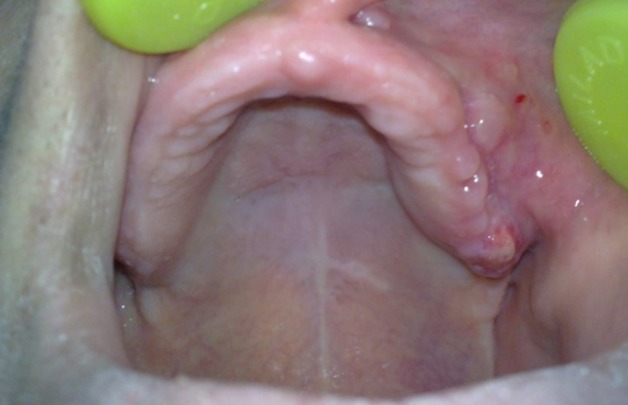
Intraoral examination after incisional biopsy showing a slight swelling of the left maxillary alveolar ridge.

Intra-oral examination revealed a firm and mild swelling overlaying the buccal and palatal aspect of the left maxillary alveolar ridge at the left premolar teeth area (Figure 1). It extended anteriorly near to the incisor teeth but did not cross the midline. In addition, the alveolar ridge was not palpable at the swelling area and loss of bone continuity was obvious.

### Radiographic Findings

**Figure 2:**
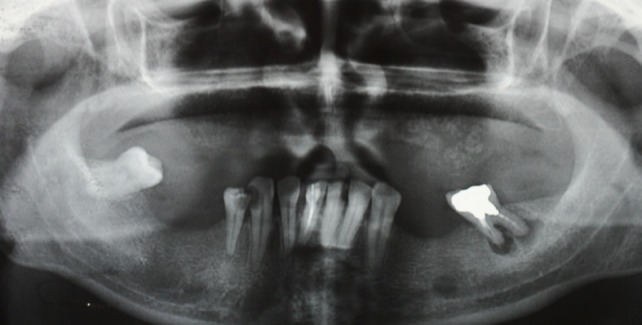
Panoramic radiograph showing an ill-defined mixed radiolucent-radiopaque lesion in the left upper jaw.

Conventional panoramic radiograph revealed a poorly defined mixed-density lesion (Figure 2). Furthermore, the occlusal view of the area demonstrated prominent bone production on the buccal surface, resembling “sunburst” appearance (not shown).

Therefore, after combining the clinical examination and radiographic evaluation, under the title of an “aggressive bone-forming neoplasm” the following differential diagnoses were proposed: 1) intramedullary osteosarcoma; 2) aggressive osteoblastoma; and 3) ossifying fibroma. Then the patient was referred to the Oral and Maxillofacial Surgery Department for an incisional biopsy.

### Incisional Biopsy Findings

**Figure 3:**
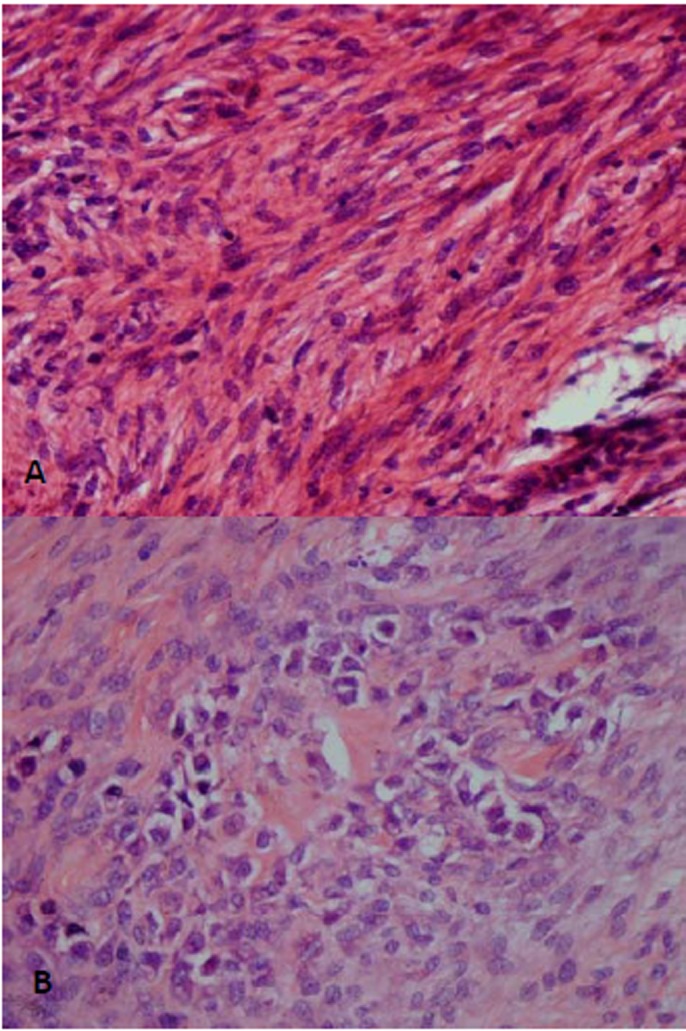
A: Photomicrograph of the incisional biopsy, showing fascicular pattern of spindle-like tumoral cells. In addition, scattered mitotic figures are seen (H&E, original magnification ×400). B: Mild pleomorphic tumoral cells, surrounded by osteoid-like eosinophilic material, are noted (H&E, original magnification ×400).

Microscopic examination of the incisional biopsy revealed heterogeneous neoplastic tissue composed of interlacing fascicles of spindle-shaped cells with plump nuclei and prominent nucleoli (Figure 3A). In addition, scattered foci of pleomorphic polygonal cells with large and hyperchromatic nuclei surrounded by amorphous eosinophilic material were noted (Figure 3B). Therefore, the first differential diagnosis of “osteosarcoma” was suggested and the patient was referred to the Oral and Maxillofacial Surgery Department for complete surgical excision.

### Surgical Resection

**Figure 4:**
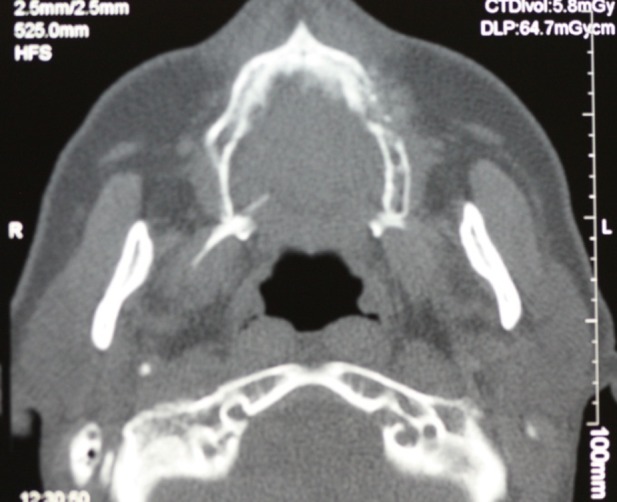
Axial CT section showing a mixed-density lesion of the left upper jaw. Destruction of the buccal bone cortex and infiltration of the tumor to the soft tissue is remarkable.

**Figure 5:**
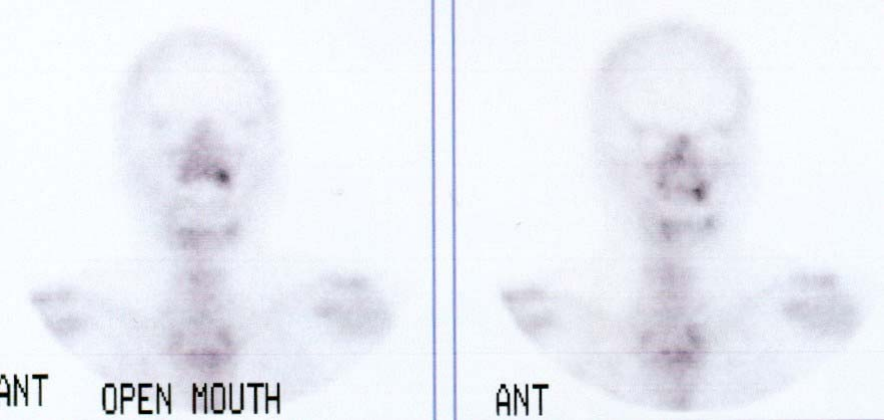
Bone scan showing an active site in the left maxilla.

**Figure 6:**
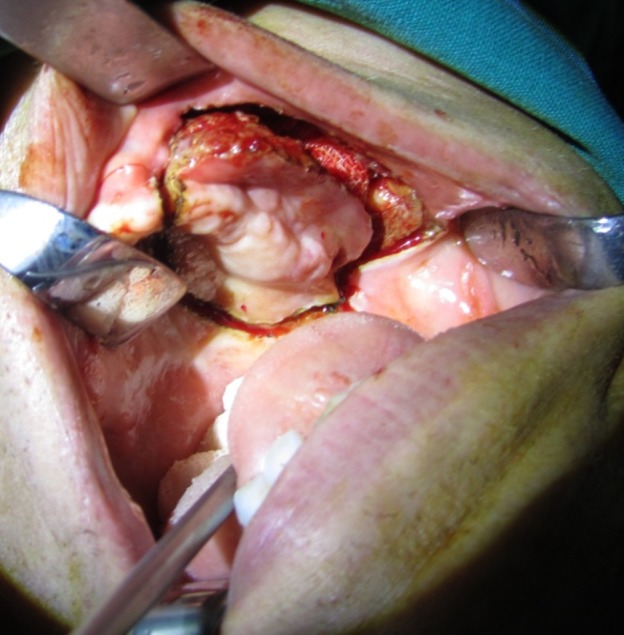
Surgical resection of the tumor with safe bony margins of 1.5 cm.

Considering the size of the lesion and its extension according to the clinical findings and radiographic evaluation on the CT scan (Figure 4), the surgeon decided to use an intraoral surgical approach to decrease the resulting morbidity from the cosmetic viewpoint. Therefore, after a whole-body bone scan with IV injection of 99m TC-MDP demonstrated focal increased activity in the left maxilla (Figure 5), complete resection of the tumor was carried out with about 1.5 cm of safe margins (Figure 6). Histopathologic examination of the fresh frozen surgical margins during surgical procedure showed no evidence of tumor involvement.

Finally, the gross surgical specimen was sent for detailed histopathologic examination. The patient was supposed to be rehabilitated occlusally and functionally afterwards, using an obturator.

### Histopathologic Findings

Histologic evaluation of the excised specimen confirmed the initial diagnosis. All the surgical margins were free of tumor.

The patient has been under close supervision clinically and radiographically for six months after the surgery and no evidence of local recurrence or distant metastasis has been observed to date.

## Discussion

OSs of the jaws are very rare and have a mild male predilection. At the time of diagnosis, the patients are approximately 2 decades older than those with extragnathic OS, which have a peak incidence between the ages of 10 and 14 years. Head and neck OSs have lower tendency to distant metastasis than their long bone counterparts, and they have a better 5-year survival rate.^4-6^

However, most Gnathic OSs are high-grade lesions^7^ and demonstrate a high mortality rate associated with local invasion that is usually difficult to control.^8^ Early diagnosis and wide surgical resection is the most important factor for prognosis, which results in a 5-year survival rate of 80%.^7^ Therefore, when the pathologic features on a biopsy specimen of the jaws are in favor of a bone-forming neoplasm, such as the current case, clinical, radiographic and histopathologic correlation is strongly recommended in establishing an accurate diagnosis.

Several histologically unusual features of OS such as epitheliod^9,10^ or small cell^11^ subtypes and variable radiographic appearances^3^ have been reported previously, which confirm the importance of meticulous evaluation of the patients with intraosseous lesions resembling OS; but a few cases with uncommon clinical presentation have been described and based on our knowledge the present case is the first reported OS with clinical presentation similar to “epulis fissurarum”. In other reported rare clinical or radiographic presentations of OS, Soares et al^2^ reported a patient with OS erroneously diagnosed as a periapical lesion and this delay in accurate diagnosis led to death of the patient after about 2 years. Yamamoto et al^12^ described a case of maxillary OS with extremely unusual image findings in an 11-year-old boy and associated this strange feature with initial phase of tumor progression. Babazade et al^13^ reported an uncommon bilateral metachronous OS of the mandibular body in a 27-year-old man and recommended that physicians should be aware that OS can occur in different sites as a true multicentric lesion.

Microscopically, the essential criterion of OS is direct production of osteoids or immature bone by tumoral cells. Three more common histologic variants including osteoblastic, chondroblastic and fibroblastic and several less common subtypes such as epithelioid and telangiectatic have been described. It seems these histopathologic subtypes do not have any great impact on the prognosis.^14^ The presence of extremely heterogeneous features makes it very difficult to diagnose OS by only interpretation of a small incisional biopsy, especially when similar, osteoblastoma-like features are encountered.^15^ In our case, excluding the radiographic findings, the fascicular pattern of spindle-shaped cells, in the absence of distinct pleomorphism and significant numbers of mitotic figures, could be easily misinterpreted as a benign mesenchymal origin spindle cell tumor such as ossifying fibroma or desmoplastic fibroma. Therefore, it is strongly recommended that the incisional biopsies of the lesions suspected to be OS should be the best and the most abundant specimens possible and conventional radiographs be requested on peripheral reactive lesions such as peripheral ossifying fibroma, pyogenic granuloma and epulis fissuratum and the final diagnosis be rendered essentially after correlation of radiographic and pathologic findings.^3^

According to several investigations the main treatment modality is still wide surgical excision and it has been demonstrated that usefulness of adjuvant chemotherapy in patients with OS of head and neck region is a matter of debate.^8^ Fernandes et al^16^ in a retrospective review of 16 gnathic OSs demonstrated no statistically significant differences in survival between patients who had received chemotherapy and those who had not been treated this way. In addition, Granowski-LeCornu et al^17^ determined improved survival in treatment of OS of the jaws in patients benefiting from improved imaging, earlier diagnosis and more aggressive treatment that included improved surgical clearance and additionally demonstrated no clear survival advantage for neo-adjuvant chemotherapy. However, it should be pointed out that some other studies have indicated that radiotherapy in addition to surgery improves local control of the tumor and survival rate of the patients significantly.^8^

## Conclusion

The authors made an attempt to verify the importance of combining the clinical, radiographic and histopathologic examinations to achieve a definitive diagnosis. It should also be emphasized that early diagnosis of OS facilitates the treatment procedure, reduces associated morbidity, significantly improves prognosis and ultimately leads to successful treatment outcome.
